# Adult-Onset Type 1 Diabetes and Pregnancy: Three Case Reports

**DOI:** 10.1155/2013/920861

**Published:** 2013-03-28

**Authors:** Barbara Bonsembiante, Maria Grazia Dalfrà, Michela Masin, Alessandra Gallo, Annunziata Lapolla

**Affiliations:** Department of Medicine, Chair of Metabolic Disease, Padova University, via Giustiniani 2, 35100 Padova, Italy

## Abstract

From 5% to 10% of diabetic patients have type 1 diabetes. Here we describe three cases of adult-onset type 1 diabetes in pregnancy treated at our clinic between 2009 and 2012. Two patients came for specialist examination during pregnancy, the third after pregnancy. These women had no prior overt diabetes and shared certain characteristics, that is, no family diabetes history, age over 35, normal prepregnancy BMI, need for insulin therapy as of the early weeks of pregnancy, and high-titer anti-GAD antibody positivity. The patients had persistent diabetes after delivery, suggesting that they developed adult-onset type 1 diabetes during pregnancy. About 10% of GDM patients become pancreatic autoantibody positive and the risk of developing overt diabetes is higher when two or more autoantibodies are present (particularly GAD and ICA). GAD-Ab shows the highest sensitivity for type 1 diabetes prediction. We need to bear in mind that older patients might conceivably develop an adult-onset type 1 diabetes during or after pregnancy. So we suggest that women with GDM showing the described clinical features shall be preferably tested for autoimmunity. Pregnant patients at risk of type 1 diabetes should be identified to avoid the maternal and fetal complications and the acute onset of diabetes afterwards.

From 5% to 10% of diabetic patients have type 1 diabetes, which results from an autoimmune destruction of pancreatic *β* cells. Autoantibodies directed against insulin, islet cells, glutamic acid decarboxylase 65 (GAD), and tyrosine phosphatase IA-2 are markers of this immune destruction [1]. Here we describe three cases of adult-onset type 1 diabetes in pregnancy treated at our clinic between 2009 and 2012.

The first concerns a 42-year-old woman who was referred for specialist examination in her 16th week of gestation (g.w.) to confirm a diagnosis of gestational diabetes. Her family history was negative for diabetes and her prepregnancy BMI was normal (22.5 kg/m^2^). Based on her glycemic profiles, insulin therapy was begun in her 17th g.w., in addition to the already-prescribed dietary restrictions. During the pregnancy, the patient's metabolic control was good (mean HbA1c 5.4%). The baby was delivered in the 39th g.w. (birth weight 3265 g). Postpartum OGTT revealed diabetes mellitus (2 h plasma glucose >200 mg/dL) with low blood insulin values (5–23.6 mU/mL), C-peptide 1.1 ng/mL, and anti-GAD Ab positivity (>2000 U/L), while HbA1c was 5.9%. In October 2010, the patient resumed insulin therapy and now has 4 injections a day, but her metabolic control remains poor (HbA1c 9.5%).

The second patient was 39 years old when she came for her first specialist examination. Her family history was negative for diabetes. Between 2004 and 2007 she had successfully completed two pregnancies, both complicated by gestational diabetes. Her prepregnancy BMI was normal (20.4 kg/m^2^). GDM had been diagnosed in the third trimester of the first pregnancy, but within the first two weeks of the second. Insulin therapy was needed in both pregnancies (right from the first few weeks in the second). Diabetes mellitus was diagnosed on postpartum OGTT after the second pregnancy. The patient took metformin therapy for several months, with no benefit. In April 2011, she was hospitalized and laboratory tests revealed: glycemia 170 mg/dL; HbA1c 7.3%, C-peptide 1.2 ng/mL; and anti-GAD Ab positivity (2000 U/L). She is currently on dietary restrictions alone.

The third patient was 36 years old and came for specialist examination in her 28th g.w. in February 2012. Her family history was negative for diabetes and her prepregnancy BMI was normal (22.4 kg/m^2^). Given her poor glycemic profiles, insulin therapy was begun immediately in addition to dietary restrictions. In the 29th g.w., blood tests revealed anti-GAD Ab positivity (268 U/L) and low fasting plasma C-peptide levels (1.8 ng/mL). During the pregnancy, the patient's metabolic control was good (mean HbA1c 6%). The baby was delivered in the 35th g.w. (birth weight 2520 g). The patient's metabolic control is currently good (HbA1c 5.6%) and she is on dietary restrictions alone.

It is important to emphasize that these women had no prior overt diabetes and they did not meet the diagnostic criteria of diabetes (i.e., FPG ≥ 126 mg/dL or 2 h plasma glucose ≥200 mg/dL during an OGTT, or HbA1c ≥6.5%, or random plasma glucose ≥200 mg/dL). Before they were referred to us for examination, all three patients had fasting plasma glucose levels below 126 mg/dL [1]. 

These pregnant women shared certain characteristics, that is, no family history of diabetes, age over 35 years, normal prepregnancy BMI, need for insulin therapy as of the early weeks of pregnancy, high-titer anti-GAD antibody positivity, and low fasting plasma C-peptide levels. The patients also had persistent diabetes after delivery, suggesting that they developed adult-onset type 1 diabetes during their pregnancy.

The literature on autoimmunity and pregnancy is limited. In most cases, GDM is characterized by a higher insulin resistance and lower insulin secretion, both of which are defects typical of type 2 diabetes. Women with GDM are consequently believed to be at higher risk of developing type 2 diabetes after pregnancy. About 10% of GDM patients become pancreatic autoantibody positive, however, so—although type 1 diabetes generally develops in younger people (under 30 years old)—we need to bear in mind that older patients might conceivably develop an adult-onset type 1 diabetes during or after pregnancy [2–4]. 

In our personal experience, these are the first cases of adult-onset type 1 diabetes occurring during pregnancy and our findings confirm the importance of identifying the risk of patients developing this type of diabetes. 

Most studies have demonstrated that the presence of diabetes-related autoimmunity in patients with GDM predicts the occurrence of type 1 diabetes; the risk of developing overt diabetes is higher when two or more autoantibodies are present (particularly GAD and ICA). Füchtenbusch et al. have reported that two years after delivery the risk was 17% with one antibody, 31% with two antibodies, and 84% with three antibodies positivity. In the study of Nilsson et al., 83% of the patients with GDM and positivity for at least one autoantibody developed type 1 diabetes 4 years after delivery. Moreover, GAD-Ab shows the highest sensitivity for type 1 diabetes prediction [5–7].

In addition, it has been found out that women with GDM and autoantibodies more frequently need insulin therapy [4, 5]; however, it has to be underlined that these patients are younger than those included in our case reports.

Up to now there are not guidelines about which patients should be checked for autoantibodies. According to our experience, we suggest that women with GDM showing clinical features as no family history of diabetes, normal pre-pregnancy BMI, need for insulin therapy in the early weeks of pregnancy, and age higher than 35 years, shall be preferably tested for autoimmunity.

Pregnant patients at risk of type 1 diabetes should be identified to avoid the maternal and fetal complications of this form of diabetes developing during the pregnancy and, very importantly, the acute onset of diabetes afterwards. Women found anti-GAD positive should be considered at high risk of developing type 1 diabetes and should consequently be regarded as potential future candidates for immunomodulatory strategies [2]. 
